# Treatment with Herbal Formula Extract in the hSOD1^G93A^ Mouse Model Attenuates Muscle and Spinal Cord Dysfunction via Anti-Inflammation

**DOI:** 10.1155/2022/4754732

**Published:** 2022-07-04

**Authors:** Eun Jin Yang, Sun Hwa Lee, Mudan Cai

**Affiliations:** ^1^KM Science Research Division, Korea Institute of Oriental Medicine (KIOM), 1672 Yuseong-daero, Yuseong-gu, Daejeon 34054, Republic of Korea; ^2^Clinical Research Division, Korea Institute of Oriental Medicine (KIOM), 1672 Yuseong-daero, Yuseong-gu, Daejeon 34054, Republic of Korea

## Abstract

Amyotrophic lateral sclerosis (ALS), a multicomplex neurodegenerative disease, has multiple underlying pathological factors and can induce other neuromuscular diseases, leading to muscle atrophy and respiratory failure. Currently, there is no effective drug for treating patients with ALS. Herbal medicine, used to treat various diseases, has multitarget effects and does not usually induce side effects. Each bioactive component in such herbal combinations can exert a mechanism of action to increase therapeutic efficacy. Herein, we investigated the efficacy of an herbal formula, comprising *Achyranthes bidentata* Blume, *Eucommia ulmoides* Oliver, and *Paeonia lactiflora* Pallas, in suppressing the pathological mechanism of ALS in male hSOD1^G93A^ mice. Herbal formula extract (HFE) (1 mg/g) were orally administered once daily for six weeks, starting at eight weeks of age, in hSOD1^G93A^ transgenic mice. To evaluate the effects of HFE, we performed footprint behavioral tests, western blotting, and immunohistochemistry to detect protein expression and quantitative PCR to detect mRNA levels in the muscles and spinal cord of hSOD1^G93A^ mice. HFE-treated hSOD1^G93A^ mice showed increased anti-inflammation, antioxidation, and regulation of autophagy in the muscles and spinal cord. Thus, HEF can be therapeutic candidates for inhibiting disease progression in patients with ALS. This study has some limitations. Although this experiment was performed only in male hSOD1^G93A^ mice, studies that investigate the efficacy of HEF in various ALS models including female mice, such as mice modeling TAR DNA-binding protein 43 (TDP43) and ORF 72 on chromosome 9 (C9*orf*72) ALS, are required before it can be established that HEF are therapeutic candidates for patients with ALS.

## 1. Introduction

Amyotrophic lateral sclerosis (ALS) is a neurodegenerative disease with various underlying pathological factors, including genetic and environmental factors that induce other neuromuscular diseases, leading to muscle atrophy and respiratory failure. The known pathological mechanisms of ALS are mitochondrial dysfunction, oxidative stress, and neuroinflammation in the glial cells [1]. Activated astrocytes and microglia release proinflammatory cytokines and toxic factors that contribute to neurotoxicity [2], and energy metabolism plays a key role in driving the onset and progression of ALS [3]. In preclinical studies, drugs have targeted these mechanisms for treating ALS; however, most drugs targeting a single process have been unsuccessful in clinical trials [4]. Therefore, there is an urgent need for an effective cure for ALS. Patients with ALS and their families have a poor quality of life. Moreover, only a few drugs, such as riluzole and edaravone, can delay death by 3–4 months. ALS results from several pathological dysfunctions, including metabolic dysfunction in the spinal cord and muscle, and therefore, new therapies and drug discoveries should be aimed at multiple targets in the muscles and spinal cord [5, 6].

Herbal medicines have been used to treat various diseases because they rarely induce side effects and have multitarget effects [7]. In ALS, conventional drugs, such as riluzole and edaravone, which are mostly ineffective and cause side effects, do not greatly prolong the survival of patients; therefore, herbal medicines have been evaluated for use in patients with ALS [8]. Each component of this herbal combination may use a different mechanism of action, thus increasing therapeutic efficacy. According to our previous study, treatment with Bojungikgi-tang showed neuroprotective effects and delayed disease progression in an ALS animal model [9]. *Achyranthes bidentata* Blume strengthens bones and muscles and protects against N-methyl-D-aspartate-induced excitotoxicity in hippocampal neurons [10] and nerve crush injury in mice [11]. *Eucommia ulmoides* Oliver improves metabolic functions by decreasing ATP levels and increasing the use of ketone bodies and glucose in the skeletal muscle [12]. *Paeonia lactiflora* Pallas exerts protective, analgesic, anti-inflammatory, and immunomodulatory effects in vitro and in vivo [13]. Since ALS is a complex disease caused by muscle dysfunction and loss of motor neurons, we examined the effects of an herbal medicine formula containing A. *bidentata* Blume, E. *ulmoides* Oliver, and P. *lactiflora* Pallas to harness the multitarget advantage of herbal medicine on tibialis anterior (TA), gastrocnemius (GC), and spinal cord (SP) of hSOD1^G93A^ mice. In this study, we demonstrated that HFE treatment exhibited anti-inflammatory and antioxidative effects and enhanced autophagy dysfunction in the TA, GC, and SP of hSOD1^G93A^ mice, suggesting that multitargeted treatment with HFE regulates the ALS-inducing pathological mechanism.

## 2. Materials and Methods

### 2.1. Animals

**Figure 1:**
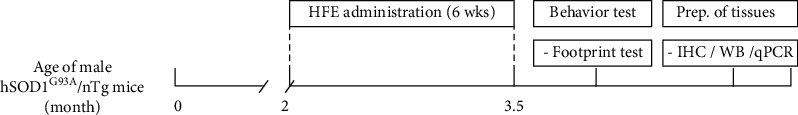
Timeline of experimental protocol. Herbal formula extract- (HFE-) treated hSOD1^G93A^ mice (Tg + HFE, *n* =8) administered 1 mg/g HFE oral injection every day for six weeks; nontransgenic (nTg, *n* =8) and transgenic (Tg, *n* =8) mice were administered distilled water. All mice were subjected to behavioral testing (footprint test) and sacrificed the following day. IHC: immunohistochemistry, WB: western blotting, qPCR: quantitative PCR.

Male B6SJL-hSOD1^G93A^ and female B6SJL mice were purchased from Jackson Laboratory (Bar Harbor, ME, USA) and mated to obtain hemizygous male hSOD1^G93A^ mice. Hemizygous hSOD1^G93A^ mice were selected as described previously [9]. Two to three mice were placed in each cage, habituated to the specific pathogen-free cages, and maintained in the specific pathogen-free animal facility at a temperature of 20 ± 2°C and humidity of 50 ± 10%, with a 12-h : 12-h light : dark cycle. Food and tap water were provided *ad libitum*. Behavioral tests were performed by experimenters who were blinded to the experimental groups. All mouse experiments were approved by the Institutional Animal Care and Use Committee of the Korea Institute of Oriental Medicine (KIOM protocol# 17-061). Male hSOD1^G93A^ mice were randomly divided into the following groups: nontransgenic mice (nTg), *n* =8; hSOD1^G93A^ transgenic mice (Tg), *n* =8; and herbal formula extract (HFE)-treated hSOD1^G93A^ transgenic mice (Tg + HFE), *n* =8. HFE (1 mg/g) was orally administered once daily for six weeks, starting at eight weeks of age in hSOD1^G93A^ transgenic mice. The nTg and hSOD1^G93A^ transgenic mice were used as controls and were administered distilled water (Figure 1).

### 2.2. Treatment with Herbal Extract

The herbal formula composed of A. *bidentata* Blume, E. *ulmoides* Oliver, and P. *lactiflora* Pallas (1 : 1 : 1) was purchased from Kwangmyungdang Medicinal Herbs Co. (Ulsan, Republic of Korea). The HFE was prepared as previously described [14].

### 2.3. Footprint Test

A footprint test was conducted on the day before the mice were sacrificed to measure motor activity. The footprint test was performed as previously described [14].

### 2.4. Tissue Preparation and Western Blotting

After anesthetizing the mice with avertin (250 mg/kg), the tibialis anterior (TA), gastrocnemius (GC), and spinal cord (SP) were collected and homogenized in RIPA buffer (50 mM Tris-Cl [pH 7.4], 1% NP-40, 0.1% sodium dodecyl sulfate [SDS], and 150 mM NaCl) containing a protease and phosphatase inhibitor cocktail (Thermo Fisher Scientific, Waltham, MA, USA). Homogenized tissues were centrifuged at 13,000 rpm for 15 min at 4°C. The total protein was quantified using a bicinchoninic acid assay kit (Pierce, Rockford, IL, USA).

Protein samples for western blotting were heated with SDS sample buffer. Equal amounts of total protein were separated on a 4–12% SDS-PAGE precast gel (Thermo Fisher Scientific) and transferred onto polyvinylidene fluoride membranes. The membranes were incubated with the specific primary antibodies for 12 h at 4°C. The primary antibodies used were anti-*β* catenin (1 : 1,000; Cell Signaling Technology, Danvers, MA, USA), anti-P62 (1 : 1,000; Cell Signaling Technology), anti-LC3b (1 : 1,000; Cell Signaling Technology), anti-GFAP (1 : 5,000; Agilent Technologies, Santa Clara, CA, USA), anti-CD11b (1 : 1,000; Abcam, Cambridge, MA, USA), anti-BAX (1 : 1,000; Santa Cruz Biotechnology, Dallas, TX, USA), anti-HO1 (1 : 1,000; Abcam), anti-ferritin (1 : 1,000; Abcam), anti-transferrin (1 : 1,000; Santa Cruz Biotechnology), anti-TGF-*β* (1 : 1,000; Cell Signaling Technology), anti-SMAD2 (1 : 1,000; Cell Signaling Technology), anti-*β*-actin (1 : 1,000; Santa Cruz Biotechnology), and Tubulin (1 : 1,000; Abcam). The membranes were then incubated with horseradish peroxidase-conjugated secondary antibodies (anti-rabbit or anti-mouse; Santa Cruz Biotechnology) and washed with TBST. The membranes were probed with enhanced chemiluminescence reagents (Thermo Fisher Scientific) and visualized using an imaging system (Bio-Rad, Hercules, CA, USA) for chemiluminescence detection. All immunoblots were quantified using the ImageJ software (National Institutes of Health, Bethesda, MD, USA).

### 2.5. Immunohistochemistry

The spinal cord was fixed in 4% paraformaldehyde and rinsed in 0.01 M PBS. Sectioned paraffin tissues were deparaffinized, and immunohistochemistry was performed as described previously [9]. The primary antibodies used were anti-CHAT (1 : 500; Thermo Scientific, Wilmington, DE, USA), anti-Iba1 (1 : 2,000; Fujifilm Wako chemical, Richmond, VA, USA), and anti-GFAP (1 : 5,000; Agilent Technologies), and the secondary antibody was an anti-horseradish peroxidase-conjugated mouse or rabbit IgG (GenDEPOT, Katy, TX, USA). The spinal cord slides were incubated with anti-mouse or rabbit IgG biotinylated secondary antibodies (Vector Laboratories, Burlingame, CA, USA) using diaminobenzidine (Vector Laboratories). Diaminobenzidine-stained slides were dehydrated with serial ethanol and xylene solutions and mounted with a coverslip and mounting solution. Images were captured using an Olympus BX51 microscope, and the intensity of the primary antibodies was quantified using ImageJ software.

### 2.6. RNA Extraction and Real-Time Reverse Transcription PCR

Total RNA was extracted from the muscle tissues using the RNA extraction kit (Intron Biotechnology, Seongnam-Si, Korea). RNA extraction and the RT-PCR were performed as previously described [15]. The primer sequences used in this study are shown in Table 1. The relative mRNA levels of the target genes were presented as fold values.

### 2.7. Statistical Analysis

All data are reported as the mean ± standard error (SEM). Statistical analyses were performed using GraphPad Prism 9 software (GraphPad, Inc., La Jolla, CA, USA). The results were analyzed with a one-way analysis of variance followed by Tukey's test for multiple comparisons. Statistical significance was set at *p* < 0.05.

## 3. Results

### 3.1. HFE Treatment Improved Motor Activity and Reduced Inflammation-Related Protein Levels in the TA and GC of hSOD1^G93A^ Mice

**Figure 2:**
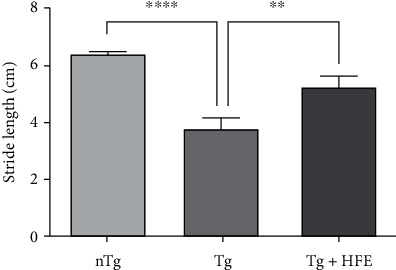
Herbal formula extract (HFE) attenuated motor function impairment. Tg mice showed decreased stride length compared with nTg mice. However, HFE treatment inhibited motor function defects in hSOD1^G93A^ mice. Data are presented as the mean ± SEM. *n* =7 mice per group. ∗∗*p* < 0.01, ∗∗∗∗*p* < 0.0001. nTg: nontransgenic mice, Tg: transgenic mice, Tg + HFE: HFE-treated transgenic mice.

To investigate the effect of HFE on motor activity, we measured the stride length of the mice in a footprint test. As shown in Figure 2, HFE improved motor function by 1.3-fold compared with the Tg group (*p* = 0.0049).

**Figure 3:**
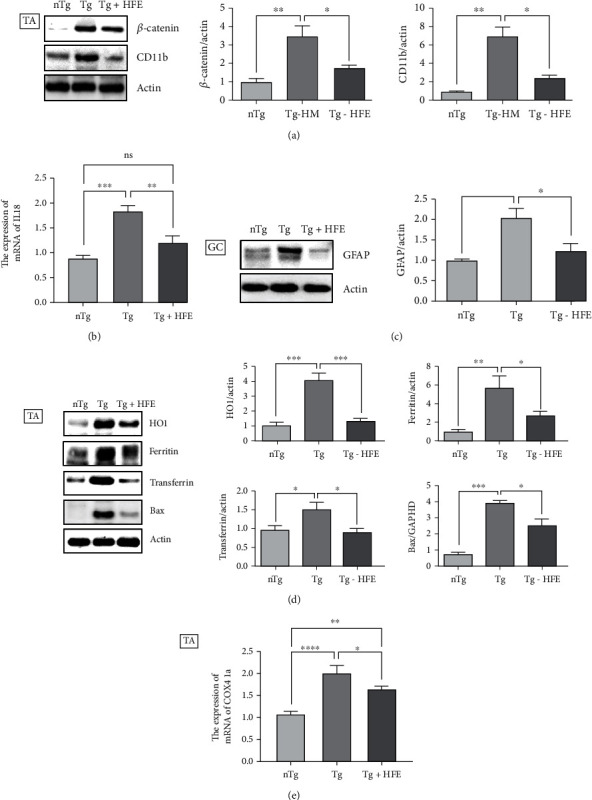
Herbal formula extract (HFE) reduced the expression of inflammatory proteins and oxidative stress-related proteins in the tibialis anterior (TA) and gastrocnemius (GC) of hSOD1^G93A^ mice. (a) Representative immunoblots of inflammatory proteins and their quantification in the TA of nontransgenic (nTg), transgenic (Tg), and HFE-Tg mice. *n* =3–4 per group. (b) mRNA expression level of IL18 in the TA of each group. *n* =4–5 mice per group. (c) Representative blot and quantification of the level of GFAP protein in the GC of nTg, Tg, and HFE + Tg mice. *n* =3–4 per group. (d) Levels of oxidative stress-related proteins (HO1, ferritin, transferrrin, and BAX) were reduced by HFE treatment in the TA of hSOD1^G93A^ mice. *n* =3–4 per group. (e) mRNA expression level of COX IV 1a in the TA of each group. *n* =4–5 mice per group. ∗*p* < 0.05, ∗∗*p* < 0.01, ∗∗∗*p* < 0.001. nTg: nontransgenic mice, Tg: transgenic mice, Tg + HFE: HFE-treated transgenic mice.

Next, to examine the biological mechanism involved in improving motor function following HFE treatment, we evaluated the effect of HFE on inflammation and oxidative stress in the TA and GC of hSOD1^G93A^ mice. Levels of inflammation-related proteins such as *β*-catenin, CD11b, and GFAP were increased by 3.4-, 6.6-, and 2.0-fold, respectively, compared with those in nTg mice (*p* = 0.0044, *p* = 0.0037, *p* = 0.0173). However, HFE treatment significantly reduced these levels by 1.9-. 2.4-, and 1.6-fold, respectively, compared with those in Tg mice (*p* = 0.0194, *p* = 0.0118, *p* = 0.0378, Figures 3(a) and 3(c)).

In addition, HFE treatment reduced the mRNA level of IL18 by 1.5-fold compared with that in the TA of Tg mice (*p* = 0.0052, Figure 3(b)). Oxidative stress is involved in inflammation [16]. Therefore, we also determined the effect of HFE on the expression levels of oxidative stress-related proteins in the TA of the nTg, Tg, and Tg-HFE groups. The levels of oxidative stress-related proteins, including HO1, ferritin, transferrin, and Bax, were increased by 4.1-, 6-, 1.5-, and 5-fold, respectively, in the TA of the Tg groups compared with those in the nTg group (*p* = 0.0004, *p* = 0.0074, *p* = 0.0468, *p* = 0.0004, Figure 3(d)). Furthermore, HFE treatment dramatically reduced HO1, ferritin, transferrin, and Bax levels in the TA of the Tg group by 3-, 2.7-, 1.6-, and 1.5-fold, respectively (*p* = 0.0006, *p* = 0.0438, *p* = 0.0223, *p* = 0.0275, Figure 3(d)). Furthermore, the mRNA level of COX IV1a was significantly reduced by 1.2-fold in the TA of HFE-treated mice compared with that in Tg mice (*p* = 0.0416, Figure 3(e)). These findings suggest that HFE treatment improved motor activity by regulating inflammatory reactions in the skeletal muscle of hSOD1^G93A^ mice.

### 3.2. HFE Treatment Regulates Autophagy Function in TA and GC of hSOD1^G93A^ Mice

**Figure 4:**
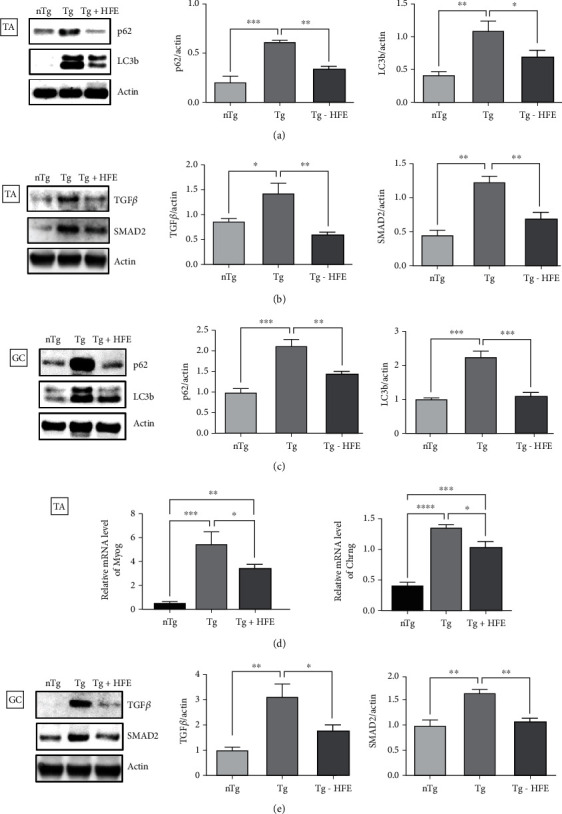
Herbal formula extract (HFE) regulated expression of autophagy- and muscle atrophy-related proteins in hSOD1^G93A^ mice. (a) Representative immunoblots and quantification of autophagy-related proteins (p62 and LC3b) and muscle atrophy-related proteins (TGF-*β* and SMAD2) in the tibialis anterior (TA) (b) and gastrocnemius (GC) (c, e) of each group. *n* =3–4 mice per group. (d) mRNA level of muscle denervation-related genes (*Myo* and *Churg*) in each group. *n* =3–5 per group. Data are presented as the mean ± SEM. ∗*p* < 0.05, ∗∗*p* < 0.01, ∗∗∗*p* < 0.001. nTg: nontransgenic mice, Tg: transgenic mice, Tg + HFE: HFE-treated transgenic mice.

Autophagy dysfunction is a pathological feature of the muscles and spinal cord in ALS. In addition, because muscle atrophy is caused by autophagy dysfunction, we investigated the expression of autophagy-related proteins, such as p62 and LC3b, and atrophy-related proteins, including TGF*β* and SMAD2, in the TA and GC of hSOD1^G93A^ mice. As shown in Figure 4, the levels of p62 and LC3b proteins were reduced by 2.0- and 1.5-fold (*p* = 0.0016, *p* = 0.0441) in the HFE-treated TA and 1.5- and 2.0-fold in the HFE-treated GC, respectively, compared with those in Tg mice (*p* = 0.0056, *p* = 0.0003, Figures 4(a) and 4(c)).

In addition, the expression of TGF*β* and SMAD2 was decreased by 2.3- and 1.7-fold (*p* = 0.0024, *p* = 0.0083) in the HFE-treated TA and 1.8- and 1.5-fold in the HFE-treated GC (*p* = 0.0413, *p* = 0.0048), respectively, compared with those in Tg mice (Figures 4(b) and 4(e)). Furthermore, HFE treatment significantly attenuated the mRNA levels of muscle denervation-related genes, myogenin (*Myog*). and cholinergic receptor nicotinic gamma subunit (*Chrng)*, by 1.5- and 1.3-fold (*p* = 0.0478, *p* = 0.025), respectively, in the TA compared with those in the Tg group (Figure 4(d)).

### 3.3. HFE Treatment Attenuates Motor Neuronal Cell Death in the Spinal Cord of hSOD1^G93A^ Mice

**Figure 5:**
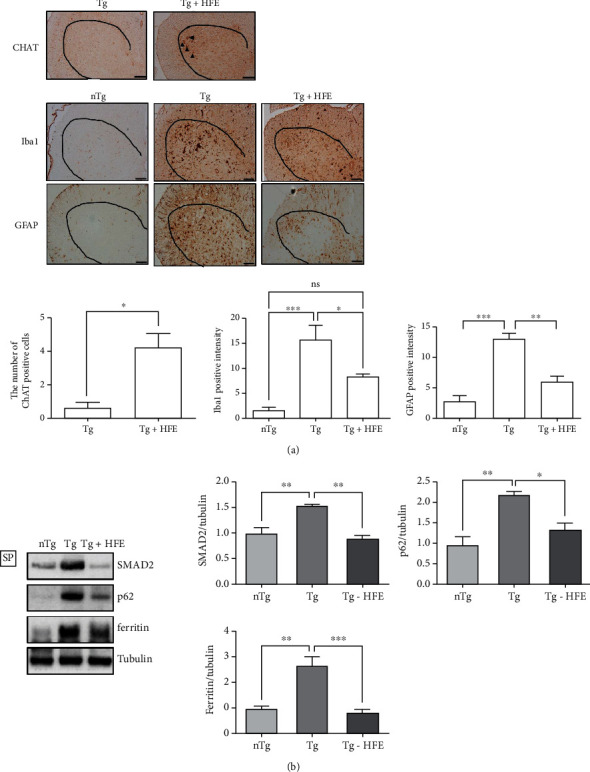
Herbal formula extract (HFE) reduced neuroinflammation in the spinal cord of hSOD1^G93A^ mice. (a) Representative images of ventral horn of spinal cord showing diaminobenzidine staining using anti-CHAT, anti-Iba1, and anti-GFAP. HFE administration increased CHAT-positive cells and reduced Iba1- and GFAP-positive cells in the ventral horn of the spinal cord in the Tg group. Quantification of positive intensity of primary antibody. *n* =3–4 mice per group. Scale bars: 2 mm. (b) Representative image of SMAD2, p62, and ferritin expression detected using western blotting analysis in the nTg, Tg, and Tg-HFE groups. Tubulin was used as the loading control. Quantification of immunoblots of GFAP, SMAD2, P62, and ferritin. Data are presented as the mean ± SEM. *n* =3–4 mice per group. ∗*p* < 0.05, ∗∗*p* < 0.01, ∗∗∗*p* < 0.001. nTg: nontransgenic mice, Tg: transgenic mice, Tg + HFE: HFE-treated transgenic mice.

To examine the effect of HFE on motor neuron death, we investigated the immunoreactivity of CHAT, Iba1, and GFAP in the spinal cord of hSOD1^G93A^ mice. The number of motor neuron cells positive for CHAT increased by 6.3-fold in the ventral horn of the spinal cord in HFE-treated mice than in hSOD1^G93A^ mice (*p* = 0.0189, Figure 5(a)). In addition, the expression of neuroinflammatory proteins, Iba1 and GFAP, was significantly reduced by 1.8- and 2.1-fold (*p* = 0.0462, *p* = 0.0012), respectively, in the spinal cord of HFE-treated hSOD1^G93A^ mice compared with that in hSOD1^G93A^ mice (Figure 5(a)). Furthermore, SMAD2, p62, and ferritin levels were dramatically decreased by 1.7-, 1.6-, and 3.2-fold (*p* = 0.0011, *p* = 0.0106, *p* = 0.0007), respectively, in the spinal cord of hSOD1^G93A^ mice following HFE treatment compared with those in Tg mice (Figure 5(b)).

## 4. Discussion

Thus far, an effective drug that can improve the quantity of life of patients with ALS is yet to be developed. Therefore, we examined the effects of herbal medicines against the multiple pathological mechanisms of ALS, including metabolic and muscle dysfunction, inflammation, autophagy dysfunction, and oxidative stress [17–19] in the muscle and spinal cord of an ALS animal model. We found that the HFE (*A. bidentata* Blume, *E. ulmoides* Oliver, and *P. lactiflora* Pallas) improved muscle function by reducing inflammation and autophagy dysfunction in the muscle and spinal cord of hSOD1^G93A^ mice.

Neuroinflammation or inflammation is critical in neurodegenerative diseases, including Alzheimer's disease, Parkinson's disease, and ALS. In ALS, microglia activation and activated astrocytes are increased in the spinal cord, inducing motor neuron death [20]. In addition, neuroinflammation in hSOD1^G93A^ mice is involved in the upregulation of NLRP3-inflammasome-related proteins [21]. However, based on these findings, most drugs target a single mechanism, such as those with antiglutamatergic (ceftriaxone, memantine, and talampanel), anti-inflammation (celecoxib, erythropoietin, and NP001), antioxidative stress, and neurotrophic effects, have failed in clinical trials [4]. TAR DNA-binding protein 43 (TARDBP, TDP-43) and ORF 72 on chromosome 9 (C9*orf*72) mutation were detected in sporadic and familiar ALS cases and involved in neuroinflammation [22, 23].

ALS is associated with muscular disease, including muscle weakness, inflammation, and denervated atrophy [24]. Specifically, inflammation and oxidative stress affect muscle homeostasis and myogenesis following FOXO activation, leading to muscle atrophy. Lawler et al. showed that muscle atrophy is induced by Foxo3 activation under oxidative stress and inflammation conditions. Huang et al. also demonstrated that oxidative stress and inflammation suppression attenuated denervation-induced muscle atrophy [25].

Autophagy is important for maintaining muscle functions; however, dysfunction in autophagy induces muscle degeneration, inflammation, oxidative stress, and mitochondrial dysfunction in ALS [26–29]. In ALS, inflammatory events in the skeletal muscle induce motor weakness, neuromuscular junction impairment, and motor neuron death. Autophagy dysfunction induces oxidative stress [30] and antioxidants through redox signaling, and the Nrf2-Keap1 pathway regulates autophagy function [31]. However, it is unclear whether autophagy activation or inactivation causes ALS. Zhang et al. reported that autophagy activation decreased the accumulation of misfolded proteins in the motor neurons of hSOD1^G93A^ mice [32]. However, Bhattacharya et al. showed that autophagy activation augmented motor neuron degeneration and did not extend the life span of hSOD1^G93A^ mice [33]. These results suggest that other mechanisms such as oxidative stress and inflammation should be considered therapeutic targets, as ALS is a complex disease. In this study, we demonstrated that HFE treatment exhibited anti-inflammatory and antioxidative effects and attenuated autophagy dysfunction in the TA, GC, and SP of hSOD1^G93A^ mice, suggesting that multitargeted treatment with HFE regulates the ALS-inducing pathological mechanism.

In the skeletal muscle, denervation-induced muscle atrophy and neuromuscular junction impairments occur with motor neuron loss, and mitochondria are involved in inducing apoptosis for denervation [34]. TGF-*β*, which causes muscle fibrosis, Smad, and histone deacetylase 4 (HDAC4), is related to ALS progression and is upregulated in ALS [35, 36]. Furthermore, HDAC4 activates the synaptic acetylcholine receptors MuSK to promote muscle reinnervation, and HDAC4 deletion contributes to neurogenic atrophy [37]. Consistently, we found that the expression of TGF-*β* and Smad proteins was increased in the muscle of hSOD1^G93A^ mice; however, treatment with HFE significantly reduced the levels of these proteins. Furthermore, denervation-related genes (*Myog* and *Chrng*) were reduced by HFE in the TA of hSOD1^G93A^ mice. Thus, treatment with HFE may prevent muscle atrophy and delay motor neuron loss in hSOD1^G93A^ mice.

Muscle metabolism is involved in body energy homeostasis, and denervation of muscle and atrophy is related to muscle metabolism. Therefore, metabolic dysfunction is a key factor in ALS. Dobrowonly et al. reported a relationship between metabolic changes and disease progression in a hSOD1^G93A^ mouse model [38] and suggested that the muscle is a critical therapeutic target in ALS. Muscle metabolism is related to mitochondrial function. Mitochondrial abnormalities with dysregulation of respiratory complexes, such as complexes I and IV, were observed in the muscles of patients with ALS and an hSOD1^G93A^ mouse model [39, 40]. We previously observed that herbal medicine improved motor activity and inhibited mitochondria cristae disruption in hSOD1^G93A^ mice [9]. Therefore, herbal medicines may improve motor function by regulating muscle metabolism via inhibition of mitochondria dysfunction in hSOD1^G93A^ mice.

## 5. Conclusions

The HFE treatment improved motor function through anti-inflammation, antioxidative, and autophagy regulation effects in the TA, GC, and SP of hSOD1^G93A^ mice. These findings suggest that HFE are therapeutic candidates for treating patients with ALS or inhibiting disease progression. However, this study has some limitations. The effects of HFE treatment on neuromuscular junction impairment in skeletal muscles and motor neurons should be further investigated in an ALS animal model. In addition, muscle metabolism and mitochondria dysfunction in hSOD1^G93A^ mice should be examined, as HFEs improve motor function. Chang et al. had reported that TDP43-induced aggregation was reduced by berberine, herbal medicine, by the regulation of mTOR-autophagy signaling pathway [41]. Therefore, studies are also needed to investigate the efficacy of HFE in various ALS models, such as mice modeling TDP43 and C9*orf*72 ALS, as this experiment was performed only in hSOD1^G93A^ mice before it can be established that HFE are therapeutic candidates for patients with ALS.

## Figures and Tables

**Table 1:** The primer sequence for real-time reverse transcription PCR.

Name	Abbreviation	Primer	Sequence (5′-3′)
Cholinergic receptor nicotinic gamma subunit	*Chrng*	F R	CTTGTGGCTAAGAAGGTGCCTG GCAAGGACACATTGAGCACGAC
Myogenin	*Myog*	F R	CCATCCAGTACATTGAGCGCCT- CTGTGGGAGTTGCATTCACTGG
Interleukin 18	*IL-18*	F R	GACAGCCTGTGTTCGAGGATATG TGTTCTTACAGGAGAGGGTAGAC
Cytochrome c oxidase subunit IV	*COX IV*	F R	CTTTTATCCTCCCAGGATTTGG GCTAAATACTTTGACACCGG
Glyceraldehyde-3-phosphate dehydrogenase	*GAPDH*	F R	CCTCGTCCCGTAGACAAA AATGAAGGGGTCGTTGATG

## Data Availability

All the data are available within the article.

## References

[1] Rossi S., Cozzolino M., Carrì M. T. (2016). Old versus new mechanisms in the pathogenesis of ALS. *Brain pathology*.

[2] Bonafede R., Mariotti R. (2017). ALS pathogenesis and therapeutic approaches: the role of mesenchymal stem cells and extracellular vesicles. *Frontiers in cellular neuroscience*.

[3] Tefera T. W., Borges K. (2017). Metabolic dysfunctions in amyotrophic lateral sclerosis pathogenesis and potential metabolic treatments. *Frontiers in neuroscience*.

[4] Petrov D., Mansfield C., Moussy A., Hermine O. (2017). ALS clinical trials review: 20 years of failure. Are we any closer to registering a new treatment?. *Frontiers in aging neuroscience*.

[5] Cook C., Petrucelli L. (2019). Genetic convergence brings clarity to the enigmatic red line in ALS. *Neuron*.

[6] Chia R., Chiò A., Traynor B. J. (2018). Novel genes associated with amyotrophic lateral sclerosis: diagnostic and clinical implications. *The Lancet Neurology*.

[7] Khan M. S. A., Ahmad I., Chattopadhyay D. (2019). *New Look to Phytomedicine : Advancements in Herbal Products as Novel Drug Leads*.

[8] Pan W., Chen X., Bao J. (2013). The use of integrative therapies in patients with amyotrophic lateral sclerosis in Shanghai, China. *Evidence-based Complementary and Alternative Medicine*.

[9] Cai M., Lee S. H., Yang E. J. (2019). Bojungikgi-tang improves muscle and spinal cord function in an amyotrophic lateral sclerosis model. *Molecular Neurobiology*.

[10] Shen H., Yuan Y., Ding F., Liu J., Gu X. (2008). The protective effects of Achyranthes bidentata polypeptides against NMDA-induced cell apoptosis in cultured hippocampal neurons through differential modulation of NR2A-and NR2B-containing NMDA receptors. *Brain research bulletin*.

[11] Yuan Y., Shen H., Yao J., Hu N., Ding F., Gu X. (2010). The protective effects of Achyranthes bidentata polypeptides in an experimental model of mouse sciatic nerve crush injury. *Brain Research Bulletin*.

[12] Fujikawa T., Hirata T., Wada A. (2010). Chronic administration of Eucommia leaf stimulates metabolic function of rats across several organs. *British Journal of Nutrition*.

[13] Zhang L., Wei W. (2020). Anti-inflammatory and immunoregulatory effects of paeoniflorin and total glucosides of paeony. *Pharmacology & Therapeutics*.

[14] Lee S. L., Cai M., Yang E. J. (2020). Anti-inflammatory Effects of a Novel Herbal Extract in the Muscle and Spinal Cord of an Amyotrophic Lateral Sclerosis Animal Model. *Front Neurosci*.

[15] Cai M., Yang E. J. Combined treatment with Bojungikgi-tang and riluzole regulates muscle metabolism and dysfunction in the hSOD1(G93A) mouse model. *Antioxidants (Basel)*.

[16] Xiong L., McCoy M., Komuro H. (2022). Inflammation-dependent oxidative stress metabolites as a hallmark of amyotrophic lateral sclerosis. *Free Radical Biology and Medicine*.

[17] Evans M. C., Couch Y., Sibson N., Turner M. R. (2013). Inflammation and neurovascular changes in amyotrophic lateral sclerosis. *Molecular and Cellular Neuroscience*.

[18] Boillée S., Velde C. V., Cleveland D. W. (2006). ALS: a disease of motor neurons and their nonneuronal neighbors. *Neuron*.

[19] Turner M. R., Bowser R., Bruijn L. (2013). Mechanisms, models and biomarkers in amyotrophic lateral sclerosis. *Amyotrophic Lateral Sclerosis and Frontotemporal Degeneration*.

[20] Burda J. E., Sofroniew M. V. (2014). Reactive gliosis and the multicellular response to CNS damage and disease. *Neuron*.

[21] Debye B., Schmülling L., Zhou L., Rune G., Beyer C., Johann S. (2018). Neurodegeneration and NLRP3 inflammasome expression in the anterior thalamus of SOD1 (G93A) ALS mice. *Brain Pathology*.

[22] Jara J. H., Gautam M., Kocak N. (2019). MCP1-CCR2 and neuroinflammation in the ALS motor cortex with TDP-43 pathology. *Journal of neuroinflammation*.

[23] Lall D., Baloh R. H. (2017). Microglia and C9orf72 in neuroinflammation and ALS and frontotemporal dementia. *The Journal of clinical investigation*.

[24] Iwasaki Y., Sugimoto H., Ikeda K., Takamiya K., Shiojima T., Kinoshita M. (1991). Muscle morphometry in amyotrophic lateral sclerosis. *International journal of neuroscience*.

[25] Huang Z., Fang Q., Ma W. (2019). Skeletal muscle atrophy was alleviated by salidroside through suppressing oxidative stress and inflammation during denervation. *Frontiers in pharmacology*.

[26] Fan Z., Xiao Q. (2020). Impaired autophagic flux contributes to muscle atrophy in obesity by affecting muscle degradation and regeneration. *Biochemical and Biophysical Research Communications*.

[27] Xiao Y., Ma C., Yi J. (2015). Suppressed autophagy flux in skeletal muscle of an amyotrophic lateral sclerosis mouse model during disease progression. *Physiological reports*.

[28] Sadeghi A., Shabani M., Alizadeh S., Meshkani R. (2020). Interplay between oxidative stress and autophagy function and its role in inflammatory cytokine expression induced by palmitate in skeletal muscle cells. *Cytokine*.

[29] Ko F., Abadir P., Marx R. (2016). Impaired mitochondrial degradation by autophagy in the skeletal muscle of the aged female interleukin 10 null mouse. *Experimental gerontology*.

[30] Lee J., Giordano S., Zhang J. (2012). Autophagy, mitochondria and oxidative stress: cross-talk and redox signalling. *Biochemical Journal*.

[31] Levonen A. L., Hill B. G., Kansanen E., Zhang J., Darley-Usmar V. M. (2014). Redox regulation of antioxidants, autophagy, and the response to stress: implications for electrophile therapeutics. *Free Radical Biology and Medicine*.

[32] Zhang X., Li L., Chen S. (2011). Rapamycin treatment augments motor neuron degeneration in SOD1G93A mouse model of amyotrophic lateral sclerosis. *Autophagy*.

[33] Bhattacharya A., Bokov A., Muller F. L. (2012). Dietary restriction but not rapamycin extends disease onset and survival of the H46R/H48Q mouse model of ALS. *Neurobiology of aging*.

[34] Siu P. M., Alway S. E. (2005). Mitochondria‐associated apoptotic signalling in denervated rat skeletal muscle. *The Journal of physiology*.

[35] Bruneteau G., Simonet T., Bauché S. (2013). Muscle histone deacetylase 4 upregulation in amyotrophic lateral sclerosis: potential role in reinnervation ability and disease progression. *Brain*.

[36] Si Y., Kim S., Cui X. (2015). Transforming growth factor beta (TGF-*β*) is a muscle biomarker of disease progression in ALS and correlates with Smad expression. *PLoS One*.

[37] Cohen T. J., Waddell D. S., Barrientos T. (2007). The histone deacetylase HDAC4 connects neural activity to muscle transcriptional reprogramming. *Journal of Biological Chemistry*.

[38] Dobrowolny G., Lepore E., Martini M. (2018). Metabolic changes associated with muscle expression of SOD1G93A. *Frontiers in physiology*.

[39] Crugnola V., Lamperti C., Lucchini V. (2010). Mitochondrial respiratory chain dysfunction in muscle from patients with amyotrophic lateral sclerosis. *Archives of neurology*.

[40] Capitanio D., Vasso M., Ratti A. (2012). Molecular signatures of amyotrophic lateral sclerosis disease progression in hind and forelimb muscles of an SOD1G93A mouse model. *Antioxidants & redox signaling*.

[41] Chang C. F., Lee Y. C., Lee K. H. (2016). Therapeutic effect of berberine on TDP-43-related pathogenesis in FTLD and ALS. *Journal of biomedical science*.

